# Development and Validation of the Nomograms for Predicting Overall Survival and Cancer-Specific Survival in Patients With Synovial Sarcoma

**DOI:** 10.3389/fendo.2021.764571

**Published:** 2022-03-04

**Authors:** Zhengqing Song, Lisha Cheng, Lili Lu, Weiqi Lu, Yuhong Zhou, Zhiming Wang

**Affiliations:** ^1^ Department of Medical Oncology, Zhongshan Hospital, Fudan University, Shanghai, China; ^2^ Department of Medical Oncology, Xiamen Branch, Zhongshan Hospital, Fudan University, Xiamen, China; ^3^ Biotherapy Centre, Zhongshan Hospital, Fudan University, Shanghai, China; ^4^ Liver Cancer Institute, Zhongshan Hospital, Fudan University, Key Laboratory of Carcinogenesis and Cancer Invasion, Ministry of Education, Shanghai, China; ^5^ Department of General Surgery, Zhongshan Hospital, Fudan University, Shanghai, China

**Keywords:** synovial sarcoma, nomogram, overall survival, cancer-specific survival, decision curve analysis

## Abstract

**Background:**

The study aimed to build and validate practical nomograms to predict overall survival (OS) and cancer-specific survival (CSS) for patients with synovial sarcoma (SyS).

**Methods:**

A total of 893 eligible patients confirmed to have SyS between 2007 and 2015 were selected from the Surveillance, Epidemiology, and End Results (SEER) database. Patients were randomly divided into the training cohort (n = 448) and validation cohort (n = 445). Clinically independent prognostic and important factors were determined according to the Akaike information criterion in multivariate Cox regression models when developing the nomograms with the training cohort. The predictive accuracy of nomograms was bootstrapped validated internally and externally with the concordance index (C-index) and calibration curve. Decision curve analysis (DCA) was performed to compare the clinical usefulness between nomograms and American Joint Commission on Cancer (AJCC) staging system.

**Results:**

Two nomograms shared common indicators including age, insurance status, tumor site, tumor size, SEER stage, surgery, and radiation, while marital status and tumor site were only included into the OS nomogram. The C-index of nomograms for predicting OS and CSS was 0.819 (0.873–0.764) and 0.821 (0.876–0.766), respectively, suggesting satisfactory predictive performance. Internal and external calibration curves exhibited optimal agreement between the nomogram prediction and the actual survival. Additionally, DCA demonstrated that our nomograms had obvious superiority over the AJCC staging system with more clinical net benefits.

**Conclusions:**

Two nomograms predicting 3- and 5-year OS and CSS of SyS patients were successfully constructed and validated for the first time, with higher predictive accuracy and clinical values than the AJCC staging system regarding OS and CSS.

## Introduction

Synovial sarcoma (SyS) is a rare malignancy that most commonly occurs in adolescents and young adults, accounting for about 6%–9% of the soft tissue sarcomas (1). SySs often originate in para-articular regions of the extremity, hardly arising within the joint (2). SySs have always been considered high-grade with particular molecular mechanism and poor prognosis (3). Due to its lower incidence, most analyses of clinical characteristics and outcome for this disease are mainly from retrospective reviews in a single center with few prospective studies available, leading to a poor understanding of this tumor. Furthermore, there still lacks a consensus of local and systemic management for SyS among clinicians, although there are multimodal approaches including surgical resection, radiotherapy, and adjuvant chemotherapy.

Because of the rarity of this tumor, to date, there is no perfect model for survival outcome prediction. Tumor-node-metastasis (TNM) staging system of the American Joint Commission on Cancer (AJCC) has long been a generally accepted formula for predicting prognosis of malignancies and represents the gold standard classification method for SyS (4). Nevertheless, a growing number of studies have demonstrated that several other factors such as age, race, tumor site and size, and non-biological factors also have an obvious impact on the prognosis of SyS patients. Additionally, the current AJCC staging system roughly divided patients into various groups but fails to evaluate the individualized survival based on patients’ demographic and clinical characteristics. Therefore, there is an urgent need to construct a novel staging system considering both patients’ status and tumor characteristics.

Prognostic nomograms are graphic and quantitative models with high precision and forecasting ability, and they have been developed in clinical practice to evaluate survival for several cancers (5–8). Compared with the AJCC staging system, nomograms can more accurately estimate survival for individual patients by integrating important prognostic variables (9). However, due to the small sample of SyS patients in each single center, no nomograms that predict overall survival (OS) or cancer-specific survival (CSS) have been developed for SyS so far.

The Surveillance, Epidemiology, and End Results (SEER) database collects the demographics, clinicopathological, and survival data of various cancer patients from population-based cancer registries in the USA, providing a favorable source to investigate rare tumors (10). In this study, we aimed to establish and validate the first comprehensive and practical SyS-targeting nomograms for OS and CSS prediction based on the SEER database. Subsequently, we comprehensively compared the performance of nomograms with that of the current AJCC staging system.

## Materials and Methods

### Patients

Patients diagnosed with SyS between 2007 and 2015 were identified from the SEER database and included in our study. The inclusion criteria were as follows: 1) International Classification of Diseases for Oncology third edition (ICD-O-3) histology code for SyS was not otherwise specified (9040/3), spindle cell (9041/3), epithelioid cell (9042/3), and biphasic (9043/3); 2) SyS was confirmed as the first and only primary malignancy by histology; 3) Patients were older than age 18 years; 4) Clinical and pathologic features were complete and detailed; 5) The follow-up was active with known outcomes. Patients whose diagnostic information could only be derived from a death certificate or autopsy report, as well as those who died within 1 month since initial diagnosis, were excluded. All the included patients were randomly allocated to the training cohort (n = 448, 50%) and validation cohort (n = 445, 50%). Institutional review board approval was not required in our study, since the SEER database is publicly available for researchers worldwide. Our accession ID to the SEER database was 10165-Nov 2017.

### Study Variables

Age, sex, race, marital status, insurance status, tumor size, pathology, histologic grade, SEER stage, chemotherapy, radiotherapy, surgery, survival months, vital status, and causes of death for each patient were extracted from the SEER database. The races included white, black, and others (American Indian/AK Native, Asian/Pacific Islander). Marital status was described as married or unmarried, while insurance status was described as Any Medicaid, insured, or uninsured. Tumor size was a continuous variable and converted to categorical variable according to optimal cutoffs, which were determined by X-tile program, a favorable software to determine optimum cut point value (tumor size, ≤6 cm, 6–10 cm, >10 cm). The tumor primary site was described as head and neck, trunk, thorax and pleura, extremities, or others. Cancer stages recorded according to the 6th AJCC stages were regrouped according to the 7th edition. OS and CSS were determined as the primary endpoints of our study. Survival time (in months) was calculated as the interval from diagnosis to death from any cause (OS) or death from SyS (CSS).

### Statistical Analysis

#### Construction of the Nomograms

The training cohort was used to build the nomograms. The univariate Cox regression analysis was used to determine factors associated with survival. Then, variables significantly associated with survival in univariate analysis were subsequently subjected to the multivariable Cox regression analysis. Finally, using the minimum value of Akaike information criterion (AIC), the backward stepwise process was used to stop rule for the multivariable Cox regression analysis and select the independent prognostic factors that strikingly contributed to patients’ survival for the constructions of the nomograms, and those factors were integrated to construct the nomograms for 3- and 5-year OS and CSS.

### Validation of the Nomograms

The validations of the nomograms were conducted both internally (training cohort) and externally (validation cohort) using C-index and calibration curve. To minimize the overfitting bias, the nomograms were subjected to 1,000 bootstrap resamples in both validations. Predictive performance was examined using the concordance index (C-index), which was analogous to the area under the curve (AUC) but more suited to censored data (11). The value of the C-index fluctuates between 0.5 (no discrimination) and 1 (perfect discrimination), and a higher C-index value means a better prognostic model (12). Calibration curves were plotted to represent the calibration between the nomogram prediction and the actual outcome. In a perfectly calibrated nomogram, the prediction would fall on a 45-degree diagonal of the calibration curve.

### Decision Curve Analysis

Decision curve analysis (DCA), a new algorithm, was performed to assess the clinical usefulness of nomograms that predict survival (13). The best nomogram would exhibit higher net clinical benefits throughout a wide range of threshold probabilities. In our study, DCA was used to compare the clinical value of the nomogram with AJCC staging system in the training and validation cohort, respectively.

All statistical analyses were performed by R software (version 3.3.0). The R packages used in our study included *rms*, *cmprsk*, *rcorrcens*, and *DecisionCurve*. All statistical tests were two-sided, and *P* value <0.05 was statistically significant.

## Results

### Patient Characteristics

**Figure 1:**
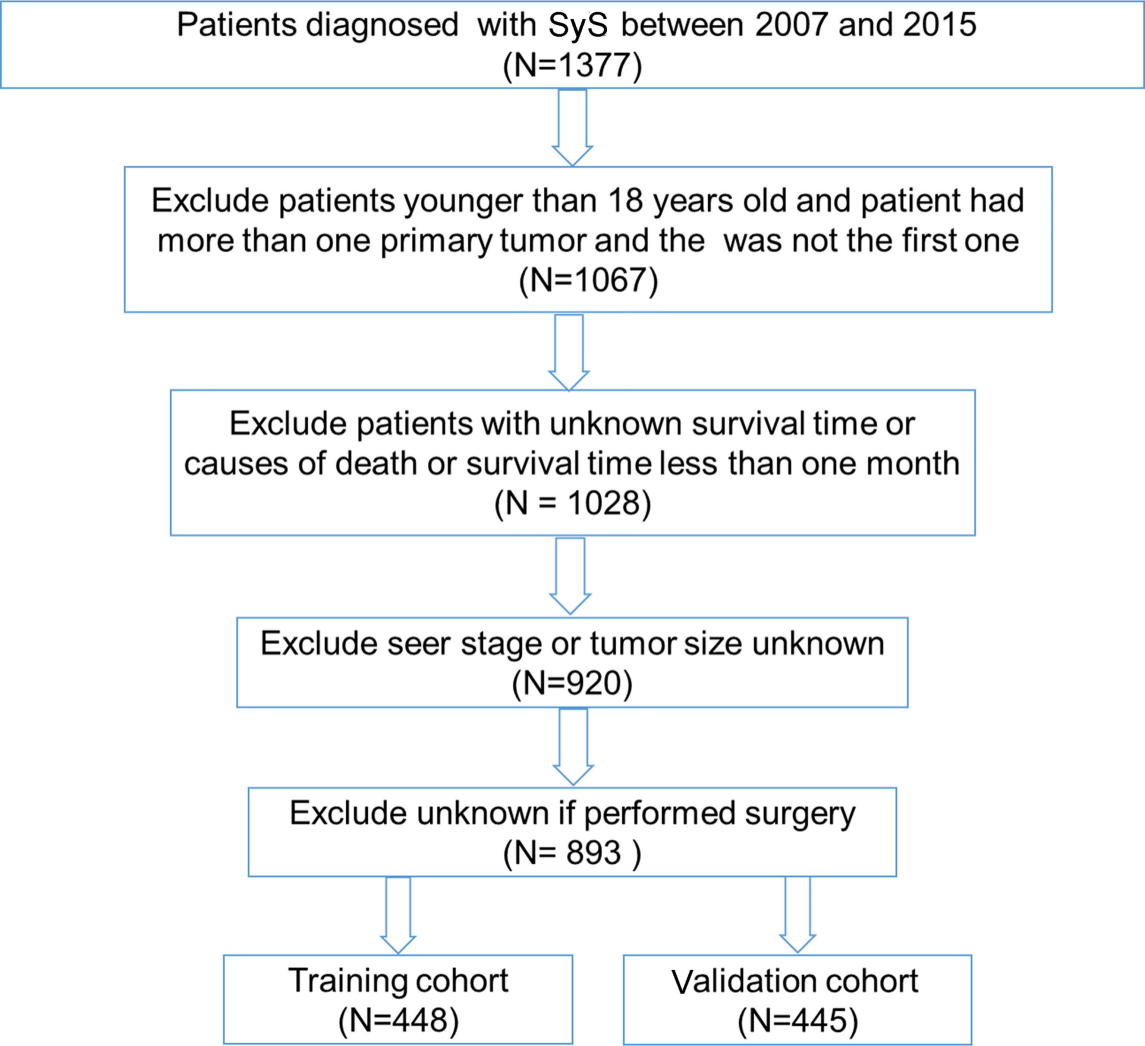
Flowchart of the synovial sarcoma (SyS) patient selection process in our study.

A total of 893 eligible SyS patients diagnosed between 2007 and 2015 in the SEER database were included in our analysis. The flowchart of the patient selection process was shown in **Figure 1**. A total of 448 and 445 of those patients were randomly allocated to the training cohort and the validation cohort, respectively. Among all the patients, the median age was 41 years with a wide range of 18–93 years. The majority of SyS patients were white (79.1%) and insured (73.3%). The most frequent tumor site was the extremities (50.1%), followed by trunk (28.9%), head and neck (13.9%), and other sites (13.2%). Regarding tumor size, ≤6 cm (43.3%) was the most frequent. Based on SEER staging, most patients (58.5%) were at SEER regional stage, 25.8% at distant stage, and 15.8% at localized stage. More than half (60.1%) of SyS patients had undergone radiotherapy, and 84.3% had received surgery. The results of a descriptive analysis about the demographic and clinicopathological characteristics were summarized in **Table 1**.

**Table 1:** Patient characteristics in the training and validation cohorts.

Characteristics	Total N (%)	Training cohort N (%)		Validation cohort N (%)
	893 (100%)		448 (50%)	445 (50%)
Age (median, range)	41 (18–93)		39.0 (18–93)	42.0 (18–93)
**Sex**				
Female	404 (45.2)		205 (45.8)	199 (44.7)
Male	489 (54.8)		243 (54.2)	246 (55.3)
**Race**				
Black	90 (10.1)		38 (8.5)	52 (11.7)
White	706 (79.1)		358 (79.9)	348 (78.2)
Others	97 (10.9)		52 (11.6)	45 (10.1)
**Marital status**				
Married	448 (50.2)		222 (49.6)	226 (50.8)
Unmarried	445 (49.8)		226 (50.4)	219 (49.2)
**Insurance status**				
Any Medicaid	160 (17.9)		72 (16.1)	88 (19.8)
Insured	655 (73.3)		341 (76.1)	314 (70.6)
Uninsured	78 (8.7)		35 (7.8)	43 (9.7)
**Tumor site**				
Head and neck	124 (13.9)		65 (14.5)	59 (13.3)
Trunk	130 (14.6)		71 (15.8)	59 (13.3)
Thorax and pleura	74 (8.3)		37 (8.3)	37 (8.3)
Extremities	447 (50.1)		219 (48.9)	228 (51.2)
Other	118 (13.2)		56 (12.5)	62 (13.9)
**Tumor size**				
≤6 cm	388 (43.4)		195 (43.5)	193 (43.4)
6–10 cm	260 (29.1)		125 (27.9)	135 (30.3)
>10 cm	245 (27.4)		128 (28.6)	117 (26.3)
**Pathology**				
Biphasic cell	169 (18.9)		96 (21.4)	73 (16.4)
Epithelioid cell	66 (7.4)		32 (7.1)	34 (7.6)
Spindle cell	278 (31.1)		143 (31.9)	135 (30.3)
NOS	380 (42.6)		177 (39.5)	203 (45.6)
**Grade**				
I	42 (4.7)		18 (4.0)	24 (5.4)
II	120 (13.4)		57 (12.7)	63 (14.2)
III	259 (29.0)		128 (28.6)	131 (29.4)
IV	169 (18.9)		89 (19.9)	80 (18.0)
Unknown	303 (33.9)		156 (34.8)	147 (33.0)
**SEER stage**				
Localized	141 (15.8)		253 (56.5)	193 (43.4)
Regional	522 (58.5)		120 (26.8)	135 (30.3)
Distant	230 (25.8)		75 (16.7)	117 (26.3)
**Chemotherapy**				
Not done	444 (49.7)		228 (50.9)	76 (17.1)
Done	449 (50.3)		220 (49.1)	369 (82.9)
**Radiotherapy**				
Not done	320 (39.9)		183 (40.8)	172 (38.9)
Done	537 (60.1)		265 (59.2)	272 (61.1)
**Surgery**				
Not done	140 (15.7)		64 (14.3)	216 (48.5)
Done	753 (84.3)		384 (85.7)	229 (51.5)

Others, American Indian/Alaska Native/Asian/Pacific Islander; NOS, not otherwise specified.

### Prognostic Nomograms for Overall Survival and Cancer-Specific Survival

**Figure 2:**
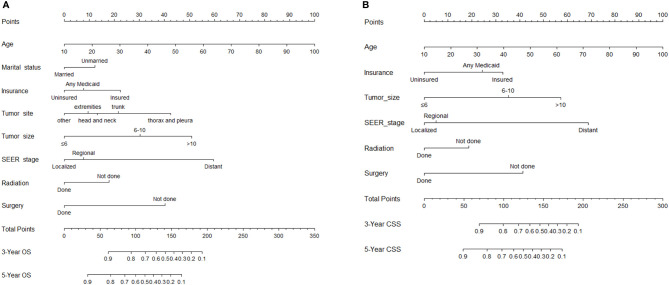
Nomogram for predicting 3- and 5-year overall survival (OS) **(A)** and cancer-specific survival (CSS) **(B)** for synovial sarcoma (SyS) patients.

In the univariate analysis, age, marital status, insurance status, pathology type, tumor site, tumor size, surgery, radiotherapy, and SEER stage were found to be significantly associated with both OS and CSS (**Table 2**). In the subsequent multivariate Cox regression, at first, all these significant factors were subjected to the Cox regression model. In order to pick out the independent prognostic factors that strikingly contributed to patients’ survival and could be admitted into the nomograms, we could take the minimum value of AIC to do the variable selection. As shown in **Table 3**, key factors for predicting OS were identified, including age, marital status, insurance status, tumor site, tumor size, SEER stage, surgery, and radiotherapy. These factors were incorporated into the nomogram for predicting the 3- and 5-year OS (**Figure 2A**). As for CSS, marital status and tumor site were ruled out from the selection (**Table 4**). Therefore, a second nomogram for predicting 3- and 5-year CSS was created using the remaining variables (**Figure 2B**).

**Table 2:** Univariate Cox regression analysis for OS and CSS of the SyS patients in the training cohort.

Characteristics	OS		CSS	
	HR (95% CI)	*P*	HR (95% CI)	*P*
**Age at diagnosis**	1.026 (1.016–1.036)	<0.001	1.023 (1.013–1.033)	<0.001
**Sex**				
Female	Reference		Reference	
Male	1.236 (0.896–1.704)	0.197	1.293 (0.923–1.812)	0.135
**Race**	0.4	0.8		
Black			Reference	
White	0.938 (0.483–1.822)	0.852	0.690 (0.408–1.167)	0.167
Others	0.938 (0.484–1.822)	0.209	1.002 (0.511–1.965)	0.996
**Marital status**				
Married	Reference		Reference	
Unmarried	1.16 (1.06–1.27)	0.002	1.411 (1.05–1.895)	0.022
**Insurance status**				
Any Medicaid	Reference		Reference	
Insured	0.496 (0.341–0.724)	<0.001	0.502 (0.339–0.743)	<0.001
Uninsured	0.607 (0.323–1.143)	0.122	0.559 (0.283–1.103)	0.093
**Tumor site**				
Head and neck	Reference		Reference	
Trunk	1.183 (0.692–2.021)	0.539	1.508 (0.846–2.687)	0.163
Thorax and pleura	2.210 (1.212–4.031)	0.009	2.658 (1.393–5.073)	0.003
Extremities	0.843 (0.527–1.349)	0.478	0.986 (0.586–1.662)	0.9604
Other	0.200 (0.081–0.491)	<0.001	0.214 (0.0793–0.576)	0.002
**Tumor size**				
≤6 cm	Reference		Reference	
6–10 cm	2.259 (1.440–3.545)	<0.001	2.549 (1.571–4.134)	<0.001
>10 cm	5.008 (3.336–7.518)	<0.001	5.706 (3.681–8.847)	<0.001
**Pathology**				
Biphasic cell	Reference		Reference	
Epithelioid cell	1.890 (1.026–3.483)	0.041	1.758 (0.922–3.357)	0.086
Spindle cell	0.878 (0.551–1.397)	0.582	0.840 (0.517–1.366)	0.015
NOS	1.761 (1.132–2.739)	0.012	1.759 (1.114–2.780)	0.482
**Grade**				
I	Reference		Reference	
II	0.434 (0.163–1.158)	0.095	0.478 (0.166–1.377)	0.172
III	0.923 (0.396–2.156)	0.853	1.062 (0.422–2.669)	0.899
IV	0.859 (0.363–2.034)	0.729	0.899 (0.351–2.306)	0.826
Unknown	0.672 (0.287–1.570)	0.358	0.739 (0.293–1.866)	0.523
**SEER stage**				
Localized	Reference		Reference	
Regional	1.875 (1.266–2.776)	0.002	1.895 (1.248–2.878)	0.003
Distant	6.918 (4.706–10.171)	<0.001	7.671 (5.141–11.448)	<0.001
**Chemotherapy**				
Not done	Reference		Reference	
Done	1.329 (0.966–1.828)	0.080	1.328 (0.952–1.854)	0.095
**Radiotherapy**				
Not done	Reference		Reference	
Done	0.6204 (0.451–0.852)	0.003	0.637 (0.456–0.888)	0.008
**Surgery**				
Not done	Reference		Reference	
Done	0.221 (0.154–0.317)	<0.001	0.212 (0.146–0.307)	<0.001

Others, American Indian/Alaska Native/Asian/Pacific Islander; NOS, not otherwise specified; OS, overall survival; CSS, cancer-specific survival; Sys, synovial sarcoma; HR, hazard ratio.

**Table 3:** Multivariate Cox regression analysis for OS of the SyS patients in the training cohort.

Characteristics	Full model		AIC-based model	
	HR (95% CI)	*P*	HR (95% CI)	*P*
**Age at diagnosis**	1.030 (1.019–1.041)	<0.001	1.033 (1.022–1.044)	<0.001
**Marital status**				
Married	Reference		Reference	
Unmarried	1.535 (1.092–2.159)	0.014	1.552 (1.114–2.164)	0.009
**Insurance status**				
Any Medicaid	Reference		Reference	
Insured	0.474 (0.244–0.921)	0.027	0.484 (0.252–0.933)	0.030
Uninsured	0.783 (0.509–1.204)	0.266	0.771 (0.505–1.177)	0.227
**Tumor site**				
Head and neck	Reference		Not selected	
Trunk	1.296 (0.866–1.937)	0.207	1.218 (0.818–1.814)	0.329
Lung and pleura	1.991 (1.276–3.104)	0.002	2.078 (1.339–3.224)	0.001
Extremities	0.775 (0.550–1.094)	0.147	0.782 (0.555–1.103)	0.161
Other	0.348 (0.197–0.614)	<0.001	0.327 (0.186–0.577)	<0.001
**Tumor size**				
≤6 cm	Reference		Reference	
6–10 cm	1.891 (1.137–3.144)	0.014	1.55 (1.35–1.77)	<0.001
>10 cm	3.735 (2.289–6.094)	<0.001	2.17 (1.89–2.47)	<0.001
**Pathology**			Not selected	
Biphasic cell	Reference			
Epithelioid cell	1.205 (0.622–2.334)	0.581	—	—
Spindle cell	0.718 (0.444–1.163)	0.466	—	—
NOS	1.195 (0.739–1.931)	0.178	—	—
**SEER stage**				
Localized	Reference		Reference	
Regional	1.088 (0.716–1.653)	0.694	1.148 (0.757–1.739)	0.514
Distant	4.734 (3.046–7.356)	<0.001	5.063 (3.289–7.792)	<0.001
**Radiotherapy**				
Not done	Reference		Reference	
Done	0.684 (0.484–0.968)	0.032	0.616 (0.439–0.862)	0.004
**Surgery**				
Not done	Reference		Reference	
Done	0.426 (0.274–0.662)	<0.001	0.366 (0.242–0.556)	<0.001

Others, American Indian/Alaska Native/Asian/Pacific Islander; NOS, not otherwise specified; AIC, Akaike information criterion; CI, confidence interval; OS, overall survival; SyS, synovial sarcoma; HR, hazard ratio.

**Table 4:** Multivariate Cox regression analysis for CSS of the SyS patients in the training cohort.

Variables	Full model		AIC-based model	
	HR (95% CI)	*P*	HR (95% CI)	*P*
**Age at diagnosis**	1.030 (1.019–1.041)	<0.001	1.0278 (1.017–1.039)	<0.001
**Marital status**			Not selected	
Married	Reference		—	—
Unmarried	1.046 (0.716–1.528)	0.817	—	—
**Insurance status**				
Any Medicaid	Reference		Reference	
Insured	0.406 (0.197–0.836)	0.015	0.442 (0.219–0.889)	0.0221
Uninsured	0.824 (0.526–1.291)	0.398	0.808 (0.528–1.235)	0.324
**Tumor site**			Not selected	
Head and neck	Reference			
Trunk	1.516 (0.816–2.816)	0.188	—	—
Lung and pleura	2.127 (1.068–4.236)	0.0316	—	—
Extremities	0.928 (0.529–1.629)	0.795	—	—
Other	0.659 (0.224–1.93)	0.448	—	—
**Tumor size**				
≤6 cm	Reference		Reference	
6–10cm	2.199 (1.269–3.809)	0.005	2.404 (1.455–3.972)	<0.001
>10 cm	4.376 (2.583–7.413)	<0.001	4.138 (2.575–6.649)	<0.001
**Pathology**			Not selected	
Biphasic cell	Reference		—	—
Epithelioid cell	1.173 (0.577–2.385)	0.659	—	—
Spindle cell	0.691 (0.411–1.162)	0.163	—	—
NOS	1.249 (0.751–2.079)	0.390	—	—
**SEER stage**				
Localized	Reference		Reference	
Regional	0.992 (0.629–1.566)	0.973	1.132 (0.729–1.757)	0.578
Distant	4.738 (2.973–7.549)	<0.001	5.503 (3.523–8.597)	<0.001
**Radiotherapy**				
Not done	Reference		Reference	
Done	0.686 (0.475–0.991)	0.044	0.629 (0.443–0.894)	0.010
**Surgery**				
Not done	Reference		Reference	
Done	0.387 (0.246–0.608)	<0.001	0.359 (0.235–0.549)	<0.001

Others, American Indian/Alaska Native/Asian/Pacific Islander; NOS, not otherwise specified; AIC, Akaike information criterion; CI, confidence interval; CSS, cancer-specific survival; SyS, synovial sarcoma; HR, hazard ratio.

### Nomogram Internal and External Validation

**Figure 3:**
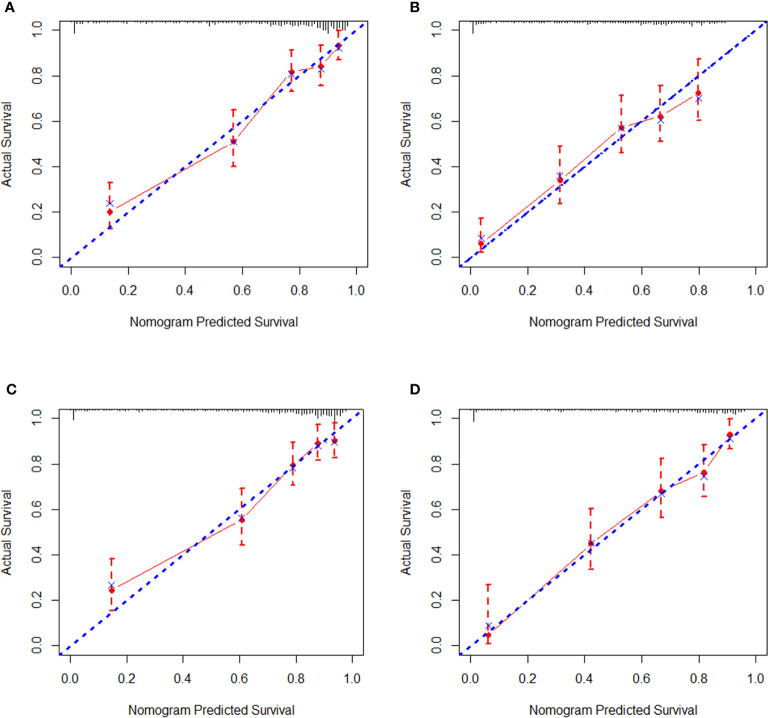
Internal calibration curves in the training cohort. **(A)** The 3-year and **(B)** 5-year overall survival (OS) nomogram calibration curves. **(C)** The 3-year and **(D)** 5-year cancer-specific survival (CSS) nomogram calibration curves.

**Figure 4:**
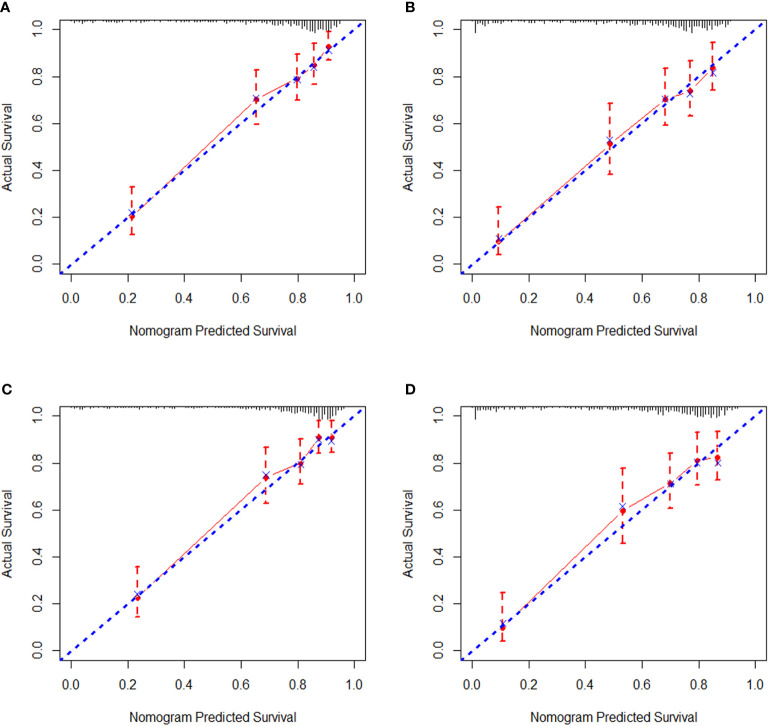
External calibration curves in the validation cohort. **(A)** The 3-year and **(B)** 5-year overall survival (OS) nomogram calibration curves. **(C)** The 3-year and **(D)** 5-year cancer-specific survival (CSS) nomogram calibration curves.

Regarding internal validation, the C-index for the nomograms to estimate OS and CSS in the training cohort was 0.819 (0.873–0.764) and 0.821 (0.876–0.766), respectively. As for external validation, the C-index for the nomograms to predict CSS and OS was 0.816 (0.865–0.767) and 0.831 (0.889–0.772), respectively. The results of C-index all demonstrated that our nomograms were suitable for SyS patients. The calibration curves of OS and CSS nomograms in the training and validation cohorts were shown in **Figures 3** and **4**, respectively, revealing optimal consistency between the prediction by our nomograms and actual survival.

Additionally, we made a comprehensive comparison between SyS nomograms for predicting OS/CSS and the current 7th AJCC staging system. In the training cohort, our nomograms yielded minimum AIC values along with maximal log-likelihoods and C-indexes for both OS and CSS compared with the AJCC stages (**Table 5** **)**, with all between-group *P* values <0.001. Similar distinction was also observed in the validation cohort. The results indicated that our nomograms had more accurate and robust predicting power than the traditional AJCC staging system.

**Table 5:** The comprehensive comparison between our nomograms and the current 7th AJCC staging system.

	Nomogram	AJCC system	*P*
**Training cohort, OS**			
AIC	1,321.357	1,362.406	—
Log-likelihood	-620.6	-677.2	<0.001
C-index (95% CI)	0.819 (0.873–0.764)	0.715 (0.765–0.664)	<0.001
**Training cohort, CSS**			
AIC	1,175.849	1,233.787	—
Log-likelihood	-575.55	-612.89	<0.001
C-index (95% CI)	0.821 (0.876–0.766)	0.726 (0.781–0.671)	<0.001
**Validation cohort, OS**			
AIC	1,212.145	1,259.111	
Log-likelihood	-593.0	-625.56	<0.001
C-index (95% CI)	0.816 (0.865–0.767)	0.731 (0.784–0.678)	<0.001
**Validation cohort, CSS**			
AIC	1,095.952	1,137.772	—
Log-likelihood	-534.98	-564.89	<0.001
C-index (95% CI)	0.831 (0.889–0.772)	0.744 (0.801–0.687)	<0.001

OS, overall survival; CSS, cancer-specific survival; AIC, Akaike information criterion; CI, confidence interval; AJCC, American Joint Commission on Cancer.

### Decision Curve Analysis

**Figure 5:**
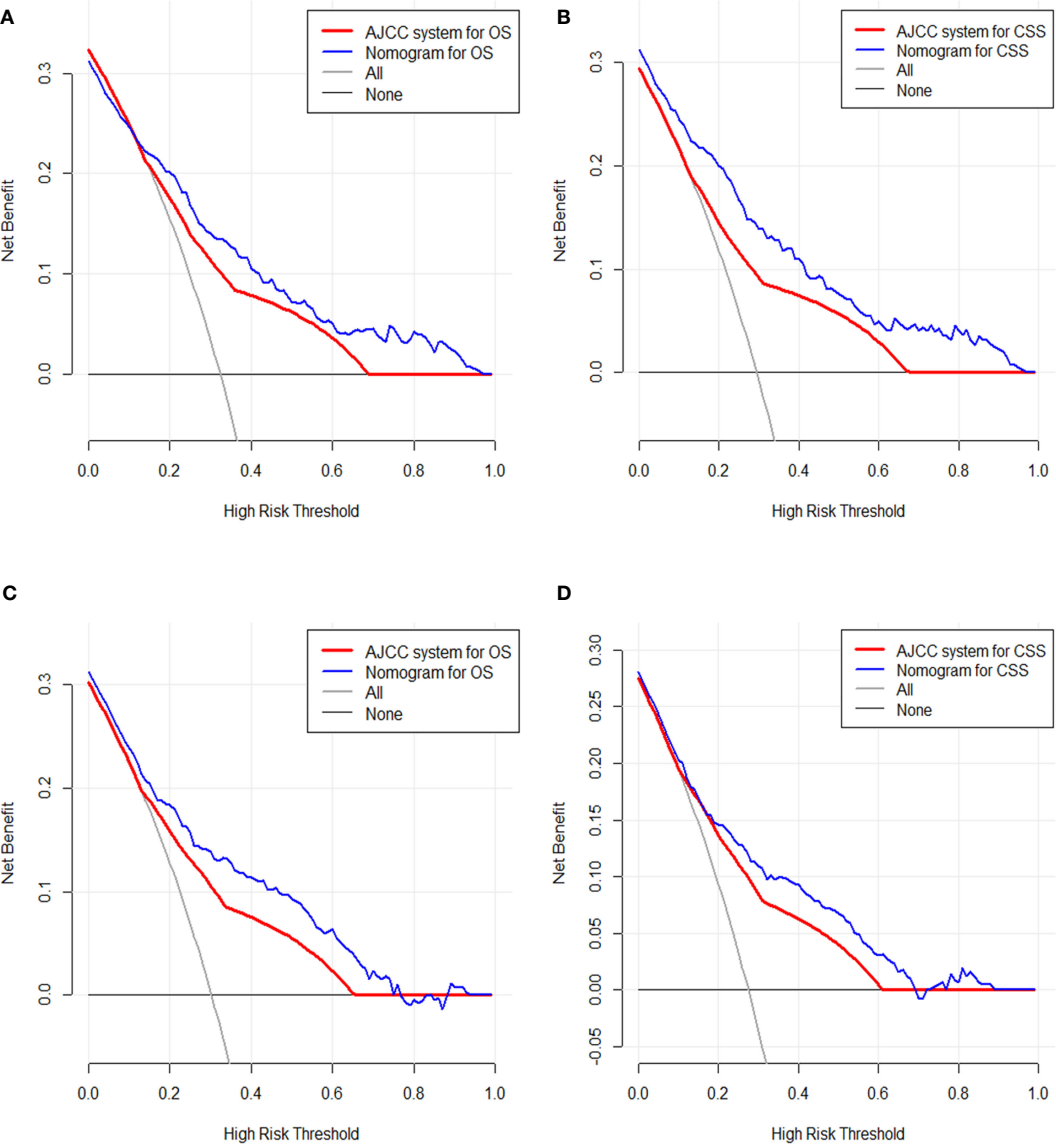
Decision curve analysis of the clinical utility between the nomograms and American Joint Commission on Cancer (AJCC) staging system regarding the overall survival (OS) **(A)** and cancer-specific survival (CSS) **(B)** in the training cohort and OS **(C)** and CSS **(D)** in the validation cohort.

After addressing the model accuracy, DCA was performed to render clinical usefulness to the nomograms using the training cohort and generalize it to the validation cohort. The nomogram had high potential for clinical application in predicting CSS and OS of SyS patients because of their wide and practical range of threshold probability through total survival of 3 or 5 years in both cohorts. When further comparing with the current AJCC staging system, our nomograms still had superiority over the AJCC staging system for the fact that more clinical net benefits were obtained in a rather wide range of threshold probabilities when using the nomograms than those when using the AJCC stages (**Figures 5A–D** **)**.

## Discussion

Due to its rarity, an accurate assessment of the prognosis for SyS remains challenging. Our knowledge of SyS is restricted to small single-center or multicenter analysis, resulting in uncertainty for the prognostic factors and optimal treatment. The SEER database provides a large sample size for researchers to identify survival-associated factors and has a greater statistical power when studying rare tumors. Herein, using the SEER database, we established the first two novel comprehensive and convenient nomograms for estimating the 3- and 5-year OS and CSS of patients diagnosed with SyS. Our nomograms exhibited satisfactory accuracy and discriminative performance in both internal and external validation. In addition, the variables in our nomograms can be easily obtained from routine clinical practice. With these nomograms, we can identify patients with different prognoses, thus facilitating individualized treatment and follow-up schedule for this rare tumor.

The nomogram has shown a wide application prospect in modern medical decision-making. It provides graphical depiction of statistical model that combines multiple parameters to calculate the probability of survival (7, 14). A number of cancer nomograms have been constructed and showed higher prediction accuracy than the current AJCC staging system, such as prostate, breast, soft tissue sarcoma, and other cancers (15), and thus it has been accepted as an alternative or even a novel staging system (16–18). To our knowledge, however, the established nomogram in our study represents the first OS and CSS nomograms for SyS that applied to the general population. Besides, higher predictive accuracy does not mean better clinical practicality. Hence, in order to overcome the limitations of the previous nomograms for other tumors, we introduced DCA in this study, and the results showed that our nomograms obtained better clinical validity and practicality with more clinical net benefits.

Recently, the impact of non-biological factors on human disease has been attached with more emphasis (19, 20). Hence, insurance and marital status were incorporated into our nomogram, which was not mentioned in all the previously reported nomograms for soft tissue sarcoma. In our analysis, we found that the insured patients had better survival OS and CSS compared with those uninsured ones. Recent studies reported that uninsured status was related to decreased diagnosis rates and increased conservative treatment for cancer patients (21), thus impairing patients’ survival. At present, the management for SyS has become prolonged, multidisciplinary, and high priced. In fact, uninsured patients usually suffer a relatively vulnerable social support network with which to tackle the challenges from SyS treatment and ultimately faced reduced access to health services and delayed admission to hospital. Just as we know, marriage is an important part of human social life, which could influence patients’ emotion, immunological function, nutrition behavior, and fit of therapy (22). And in our analysis, marital status was demonstrated to be an independent prognostic factor for OS. This result has been confirmed in various kinds of cancers (23–25). The married patients tend to enjoy good psychological state, healthy lifestyles, and sound social support networks (26), and this could contribute to their survival advantages to a large extent. Taken together, we strongly recommend integration of non-biological factors into the prognosis prediction system for cancer patients.

Generally speaking, our study has several advantages in the following aspects. First, no prognostic nomogram has been established for SyS patients before. We established the first two nomograms for these patients and made the individualized prediction of prognosis become possible. Furthermore, our nomogram showed better discriminating power in predicting OS and CSS than the SEER and 7th edition AJCC staging system did. Second, our nomograms were based on a larger-scale population than the SEER database, which provided rich and detailed data. Actually, sufficient samples incorporated are necessary for the accuracy of nomograms. Third, simplicity and user-friendliness were a strength of our nomogram. We used the AIC to minimize the number of parameters used in the nomograms, and these parameters were easily available and measurable for clinicians. Fourth, as we mentioned above, it was the first to reveal that non-biological factors including marital status and insurance status were independent prognostic factors for SyS patients and were incorporated into our nomograms for OS and CSS prediction. Last but not least, DCA, a novel method for analyzing clinical usefulness, was introduced in our nomograms and showed that the new nomograms had wider clinical applicability than the current AJCC staging system.

Inevitably, our study had several limitations that should be noted. The nomograms were established using retrospective data from the SEER database, which may introduce several unavoidable biases, such as treatment selection bias and missing data. Second, the several important prognostic factors of soft tissue sarcoma that were determined in previous studies, such as performance status score, comorbidity, the usage of mammalian target of rapamycin (mTOR) inhibitors or anti-angiogenic agents, and the detailed information of chemotherapy and surgery, were not taken into consideration in our study, since they were unavailable in the SEER database. Third, there was no other independent database available to validate our nomograms externally, hence we used the same retrospective dataset to establish and validate the nomograms. As we know, external validation with independent data was required to evaluate whether it was applicable for another patient groups. And to further refine our nomograms, prospective validation with independent patients was warranted.

In conclusion, for patients with SyS, we developed and validated the first two nomograms that estimated 3- and 5-year OS and CSS by using population-based data. These nomograms showed more accurate predictive performance and clinical usefulness than the AJCC staging system for predicting CSS and OS. However, performing further external valuation with other independent patients is still warranted.

## Data Availability Statement

The raw data supporting the conclusions of this article will be made available by the authors without undue reservation.

## Ethics Statement

The experiments were approved by the Ethics Committee of Zhongshan Hospital, Fudan University.

## Author Contributions

ZW and YZ: conceptualization, investigation, methodology, project administration, writing—review, editing, and supervision. ZS and LC: data curation, formal analysis, investigation, methodology, and writing—review. LL and WL: methodology, validation, writing—review, and editing. All authors have read and approved the article.

## Funding

The present study was supported by the Science and Technology Planning Project of Xiamen, Fujian Province, China (No.3502Z20199127, 3502Z20214ZD1061).

## Conflict of Interest

The authors declare that the research was conducted in the absence of any commercial or financial relationships that could be construed as a potential conflict of interest.

## Publisher’s Note

All claims expressed in this article are solely those of the authors and do not necessarily represent those of their affiliated organizations, or those of the publisher, the editors and the reviewers. Any product that may be evaluated in this article, or claim that may be made by its manufacturer, is not guaranteed or endorsed by the publisher.

## References

[1] Arvind R Radhika S. And Gautam U Malignant Small Round Cell Tumors. J Cytol (2009) 26(1):1–10. doi: 10.4103/0970-9371.54861 21938141 PMC3167982

[2] Khin T. And Cyril F Synovial Sarcoma: Defining Features and Diagnostic Evolution. Ann Diagn Pathol (2014) 18(6):369–80. doi: 10.1016/j.anndiagpath.2014.09.002 25438927

[3] Mallen-St Clair J Arshi A Abemayor E St John M. Factors Associated With Survival in Patients With Synovial Cell Sarcoma of the Head and Neck: An Analysis of 167 Cases Using the SEER (Surveillance, Epidemiology, and End Results) Database. JAMA Otolaryngol Head Neck Surg (2016) 142(6):576–83. doi: 10.1001/jamaoto.2016.0384 PMC6173585 27100936

[4] Cates JMM. The AJCC 8th Edition Staging System for Soft Tissue Sarcoma of the Extremities or Trunk: A Cohort Study of the SEER Database. J Natl Compr Canc Netw (2018) 16(2):144–52. doi: 10.6004/jnccn.2017.7042 29439175

[5] Wakai K Utsumi T Yoneda K Oka R Endo T Yano M. Development and External Validation of a Nomogram to Predict High-Grade Papillary Bladder Cancer Before First-Time Transurethral Resection of the Bladder Tumor. Int J Clin Oncol (2018) 23(5):957–64. doi: 10.1007/s10147-018-1299-y 29804156

[6] Wang F Zhang H Wen J Zhou J Liu Y Cheng B. Nomograms Forecasting Long-Term Overall and Cancer-Specific Survival of Patients With Oral Squamous Cell Carcinoma. Cancer Med (2018) 7(4):943–52. doi: 10.1002/cam4.1216 PMC5911576 29512294

[7] Touijer K. And Scardino PT Nomograms for Staging, Prognosis, and Predicting Treatment Outcomes. Cancer (2009) 115(13 Suppl):3107–11. doi: 10.1002/cncr.24352 19544538

[8] Iasonos A Schrag D Raj GV Panageas KS. How to Build and Interpret a Nomogram for Cancer Prognosis. J Clin Oncol (2008) 26(8):1364–70. doi: 10.1200/JCO.2007.12.9791 18323559

[9] Nieder C Mehta MP Geinitz H Grosu AL. Prognostic and Predictive Factors in Patients With Brain Metastases From Solid Tumors: A Review of Published Nomograms. Crit Rev Oncol Hematol (2018) 126:13–8. doi: 10.1016/j.critrevonc.2018.03.018 29759555

[10] Cronin KA Ries LA. And Edwards BK the Surveillance, Epidemiology, and End Results (SEER) Program of the National Cancer Institute. Cancer (2014) 120(Suppl 23):3755–7. doi: 10.1002/cncr.29049 25412387

[11] Harrell F Lee K. And Mark D Multivariable Prognostic Models: Issues in Developing Models, Evaluating Assumptions and Adequacy, and Measuring and Reducing Errors. Stat Med (1996) 15(4):361–87. doi: 10.1002/(SICI)1097-0258(19960229)15:4<361::AID-SIM168>3.0.CO;2-4 8668867

[12] Wolbers M Koller MT Witteman JC Steyerberg EW. Prognostic Models With Competing Risks: Methods and Application to Coronary Risk Prediction. Epidemiology (2009) 20(4):555–61. doi: 10.1097/EDE.0b013e3181a39056 19367167

[13] Van Calster B Wynants L Verbeek JFM Verbakel JY Christodoulou E Vickers AJ. Reporting and Interpreting Decision Curve Analysis: A Guide for Investigators. Eur Urol (2018) 74(6):796–804. doi: 10.1016/j.eururo.2018.08.038 30241973 PMC6261531

[14] Bianco FJ Jr. Nomograms and Medicine. Eur Urol (2006) 50(5):884–6. doi: 10.1016/j.eururo.2006.07.043 16973258

[15] Fisher SB Chiang YJ Feig BW Cormier JN Hunt KK Torres KE. Comparative Performance of the 7th and 8th Editions of the American Joint Committee on Cancer Staging Systems for Soft Tissue Sarcoma of the Trunk and Extremities. Ann Surg Oncol (2018) 25(5):1126–32. doi: 10.1245/s10434-018-6378-9 PMC6135610 29468609

[16] Chen J Fang A Chen M Tuoheti Y Zhou Z Xu L. A Novel Inflammation-Based Nomogram System to Predict Survival of Patients With Hepatocellular Carcinoma. Cancer Med (2018) 7(10):5027–35. doi: 10.1002/cam4.1787 PMC6198220 30259688

[17] Kong X Li J Cai Y Tian Y Chi S Tong D. A Modified TNM Staging System for Non-Metastatic Colorectal Cancer Based on Nomogram Analysis of SEER Database. BMC Cancer (2018) 18(1):50. doi: 10.1186/s12885-017-3796-1 29310604 PMC5759792

[18] Pan JJ Ng WT Zong JF Lee SW Choi HC Chan LL. Prognostic Nomogram for Refining the Prognostication of the Proposed 8th Edition of the AJCC/UICC Staging System for Nasopharyngeal Cancer in the Era of Intensity-Modulated Radiotherapy. Cancer (2016) 122(21):3307–15. doi: 10.1002/cncr.30198 PMC5524130 27434142

[19] Jakobsen L Niemann T Thorsgaard N Thuesen L Lassen JF Jensen LO. Dimensions of Socioeconomic Status and Clinical Outcome After Primary Percutaneous Coronary Intervention. Circ Cardiovasc Interv (2012) 5(5):641–8. doi: 10.1161/CIRCINTERVENTIONS.112.968271 23031837

[20] Shapiro M Chen Q Huang Q Boosalis VA Yoon CH Saund MS. Associations of Socioeconomic Variables With Resection, Stage, and Survival in Patients With Early-Stage Pancreatic Cancer. JAMA Surg (2016) 151(4):338–45. doi: 10.1001/jamasurg.2015.4239 26581025

[21] Deb S Pendharkar AV Schoen MK Altekruse S Ratliff J Desai A. The Effect of Socioeconomic Status on Gross Total Resection, Radiation Therapy and Overall Survival in Patients With Gliomas. J Neurooncol (2017) 132(3):447–53. doi: 10.1007/s11060-017-2391-2 28258423

[22] Aizer AA Chen MH McCarthy EP Mendu ML Koo S Wilhite TJ. Marital Status and Survival in Patients With Cancer. J Clin Oncol (2013) 31(31):3869–76. doi: 10.1200/JCO.2013.49.6489 PMC4878087 24062405

[23] Xie JC Yang S Liu XY Zhao YX. Effect of Marital Status on Survival in Glioblastoma Multiforme by Demographics, Education, Economic Factors, and Insurance Status. Cancer Med (2018) 7(8):3722–42. doi: 10.1002/cam4.1688 PMC6089174 30009575

[24] Shi RL Qu N Lu ZW Liao T Gao Y Ji QH. The Impact of Marital Status at Diagnosis on Cancer Survival in Patients With Differentiated Thyroid Cancer. Cancer Med (2016) 5(8):2145–54. doi: 10.1002/cam4.778 PMC4898978 27264532

[25] Costa LJ Brill IK. And Brown EE Impact of Marital Status, Insurance Status, Income, and Race/Ethnicity on the Survival of Younger Patients Diagnosed With Multiple Myeloma in the United States. Cancer (2016) 122(20):3183–90. doi: 10.1002/cncr.30183 27548407

[26] Chin B Murphy MLM Janicki-Deverts D Cohen S. Marital Status as a Predictor of Diurnal Salivary Cortisol Levels and Slopes in a Community Sample of Healthy Adults. Psychoneuroendocrinology (2017) 78:68–75. doi: 10.1016/j.psyneuen.2017.01.016 28171850 PMC5365082

